# Clinicopathological significance of non-small cell lung cancer with high prevalence of Oct-4 tumor cells

**DOI:** 10.1186/1756-9966-31-10

**Published:** 2012-02-02

**Authors:** Zhenguang Chen, Tao Wang, Lie Cai, Chunhua Su, Beilong Zhong, Yiyan Lei, Andy Peng Xiang

**Affiliations:** 1 Department of Thoracic Surgery, The First Affiliated Hospital, Sun Yat-sen University, Lung Cancer Research Center of Sun Yat-sen University, Guangzhou, Guangdong 510080, China; 2 Center for Stem Cell Biology and Tissue Engineering, Sun Yat-sen University, Key Laboratory for Stem Cells and Tissue Engineering, Ministry of Education, Guangzhou, Guangdong 510080, China; 3 Department of Rehabilitation, The First Affiliated Hospital, Sun Yat-sen University, Guangzhou, Guangdong 510080, China; 4 Department of Thoracic Surgery, The Fifth Affiliated Hospital, Sun Yat-sen University, Zhuhai, Guangdong 519000, China

**Keywords:** Oct-4, Non-small cell lung cancer, Prognosis, Proliferation, Angiogenesis

## Abstract

**Background:**

Expression of the stem cell marker octamer 4 (Oct-4) in various neoplasms has been previously reported, but very little is currently known about the potential function of Oct-4 in this setting. The purpose of this study was to assess the prognostic value of Oct-4 expression after surgery in primary non-small cell lung cancer (NSCLC) and investigate its possible molecular mechanism.

**Methods:**

We measured Oct-4 expression in 113 NSCLC tissue samples and three cell lines by immunohistochemical staining and RT-PCR. The association of Oct-4 expression with demographic characteristics, proliferative marker Ki67, microvessel density (MVD), and expression of vascular endothelial growth factor (VEGF) were assessed.

**Results:**

Oct-4 expression was detected in 90.3% of samples and was positively correlated with poor differentiation and adenocarcinoma histology, and Oct-4 mRNA was found in each cell lines detected. Overexpression of Oct-4 had a strong association with cells proliferation in all cases, MVD-negative, and VEGF-negative subsets. A Kaplan-Meier analysis showed that overexpression of Oct-4 was associated with shorter overall survival in all cases, adenocarcinoma, squamous cell carcinoma, MVD-negative, and VEGF-negative subsets. A multivariate analysis demonstrated that Oct-4 level in tumor tissue was an independent prognostic factor for overall survival in all cases, MVD-negative, and VEGF-negative subsets.

**Conclusion:**

Our findings suggest that, even in the context of vulnerable MVD status and VEGF expression, overexpression of Oct-4 in tumor tissue represents a prognostic factor in primary NSCLC patients. Oct-4 may maintain NSCLC cells in a poorly differentiated state through a mechanism that depends on promoting cell proliferation.

## Background

Despite recent progress in treatment, lung cancer remains the leading cause of cancer deaths in both women and men throughout the world [1]. Not all patients with lung cancer benefit from routine surgery and chemotherapy. This is especially true for those with primary non-small cell lung cancer (NSCLC), the most common malignancy in the thoracic field, where such therapies have been tried with limited efficacy [2]. To improve patient survival rate, researchers have increasingly focused on understanding specific characteristics of NSCLCs as a means to elucidate the mechanism of tumor development and develop possible targeted therapeutic approaches.

Octamer 4 (Oct-4), a member of the POU-domain transcription factor family, is normally expressed in both adult and embryonic stem cells [3,4]. Recent reports have demonstrated that Oct-4 is not only involved in controlling the maintenance of stem cell pluripotency, but is also specifically responsible for the unlimited proliferative potential of stem cells, suggesting that Oct-4 functions as a master switch during differentiation of human somatic cell [5-7]. Interestingly, Oct-4 is also re-expressed in germ cell tumors [8], breast cancer [9], bladder cancer [10], prostate cancer and hepatomas [11,12], but very little is known about its potential function in malignant disease [13]. Moreover, overexpression of Oct-4 increases the malignant potential of tumors, and downregulation of Oct-4 in tumor cells inhibits tumor growth, suggesting that Oct-4 might play a key role in maintaining the survival of cancer cells [13,14]. Although its asymmetric expression may indicate that Oct-4 is a suitable target for therapeutic intervention in adenocarcinoma and bronchioloalveolar carcinoma [15], the role of Oct-4 expression in primary NSCLC has remained ill defined.

To address this potential role, we assessed Oct-4 expression in cancer specimens from 113 patients with primary NSCLC by immunohistochemical staining. We further investigated the association of Oct-4 expression in NSCLC tumor cells with some important clinical pathological indices. In addition, we examined the involvement of Oct-4 in tumor cell proliferation and tumor-induced angiogenesis in NSCLC by relating Oct-4 expression with microvessel density (MVD), and expression of Ki-67 and vascular endothelial growth factor (VEGF), proliferative and the vascular markers, respectively. On the basis of previous reports that a subset of NSCLC tumors do not induce angiogenesis but instead co-opt the normal vasculature for further growth [16,17], we also evaluated associations of Oct-4 expression with tumor cell proliferation and prognosis in subsets of patients with weak VEGF-mediated angiogenesis (disregarding the nonangiogenic subsets of NSCLC in the analysis, which would tend to obscure the role of Oct-4 expression in primary NSCLC).

Our results provide the first demonstration that expression of the stem cell marker Oct-4 maintains tumor cells in a poorly differentiated state through a mechanism that depends on promoting cell proliferation. Moreover, even in the context of vulnerable MVD status and VEGF expression, Oct-4 plays an important role in tumor cell proliferation and contributes to poor prognosis in human NSCLC.

## Methods

### Patients and tissue specimens

Cancer tissue and corresponding adjacent normal tissue (within 1-2 cm of the tumor edge) from 113 primary NSCLC cases were randomly selected from our tissue database. Patients had been treated in the Department of Thoracic Surgery of the First Affiliated Hospital of Sun Yat-sen University from Jan 2003 to July 2004. None of the patients had received neoadjuvant chemotherapy or radiotherapy. Clinical information was obtained by reviewing the perioperative medical records, or by telephone or written correspondence. Cases were staged according to the tumor-node-metastases (TNM) classification of the International Union Against Cancer, revised in 2002 [18]. The study was approved by the Medical Ethical Committee of the First Affiliated Hospital, Sun Yat-sen University. Paraffin-embedded specimens of each case were sectioned and fixed on siliconized slides. Histological typing was determined according to World Health Organization classifications [19]. Tumor size and metastatic lymph node number and locations were obtained from pathology reports.

### Cell lines

The primary NSCLC cell lines, A549, H460 and H1299, obtained from the Cell Bank of the Chinese Academy of Science (Shanghai, China), were cultured in RPMI 1640 medium (Gibco/Invitrogen, Camarillo, CA, USA) supplemented with 10% fetal bovine serum (Hyclone, Logan, UT, USA).

### Immunohistochemical staining and evaluation

The primary antibodies used in this study were as follow: anti-Oct-4 (sc-5279, dilution 1:100; Santa Cruz Biotechnology, Santa Cruz, CA, USA), anti-Ki-67 (ab92742, dilution 1:200; Abcam, Cambridge, UK), and anti-VEGF (sc-7269, dilution 1:100; Santa Cruz Biotechnology, Santa Cruz, CA, USA). Immunohistochemical staining was carried out using the streptavidin-peroxidase method. Cells with nuclear staining for Oct-4 and Ki-67, and cytoplasmic staining for VEGF, were scored as positive for the respective marker. The intensity of Oct-4, Ki-67, and VEGF staining was scored on a 0-to-3 scale: 0, negative; 1, light; 2, moderate; and 3, intense. The percentage of the tumor area stained for each marker at each intensity was calculated by dividing the number of tumor cells positive for the marker at each intensity by the total number of tumor cells. Areas that were negative were given a value of 0. A total of 10-12 discrete foci in each section were examined microscopically (400× magnification) to generate an average staining intensity and percentage of the surface area covered. The final histoscore was calculated using the formula: [(1 × percentage of weakly positive tumor cells) + (2 × percentage of moderately positive tumor cells) + (3 × percentage of intense positive tumor cells)]. The median values of Oct-4, Ki-67, and VEGF histoscores were used to classify samples as positive (above the median) or negative (below the median) for each marker.

### Evaluation of MVD

Immunohistochemical staining for CD34 (MS-363, dilution 1:50; Lab Vision, Fremont, CA; Clone QBEnd/10) was analyzed. After identifying the three most vascularized areas within a tumor ("hot spots") at low magnification (×40), vessels in three representative fields in each of these areas were counted at high magnification (×400; 0.152 mm^2^; 0.44 mm diameter). The high-magnification fields were then marked for subsequent image cell counting analysis. Single immunoreactive endothelial cells or endothelial cell clusters separated from other microvessels were counted as individual microvessels. Endothelial staining in large vessels with tunica media and nonspecific staining of non-endothelial structures were excluded from microvessel counts. The mean visual microvessel density for CD34 was calculated as the average of six counts (three hot spots and three microscopic fields). Microvessel counts greater than the median counts were taken as MVD-positive, and microvessel counts lower than the median were taken as MVD-negative.

### Reverse transcription-polymerase chain reaction (RT-PCR)

Total RNA was extracted from cultured cells using the TRIzol reagent (Invitrogen, Grand Island NY, USA), according to the manufacturer's instructions. Extracted RNA was treated with DNase (Fermentas, Vilnius, Lithuania) to remove DNA contamination. For cDNA synthesis, 1 μg of total RNA was reverse transcribed using a RevertAid First Strand cDNA Synthesis Kit (Fermentas). PCR was performed with ExTaq (TaKaRa, Japan). The primer sequences and sizes of amplified products were as follows: Oct-4, 5'-GAC AGG GGG AGG GGA GGA GCT AGG-3' and 5'-CTT CCC TCC AAC CAG TTG CCC CAA AC-3' (142 bp); β-actin (internal control), 5'-GTG GGG CGC CCC AGG CAC CA-3' and 5'-CTC CTT AAT GTC ACG CAC GAT TTC-3' (540 bp).

### Statistical analysis

All calculations were done using SPSS V.14.0 software (Chicago, IL, USA). Spearman's coefficient of correlation, Chi-squared tests, and Mann-Whitney tests were used as appropriate. A multivariate model was used to evaluate statistical associations among variables. A Cox regression model was used to relate potential prognostic factors with survival.

## Results

### Basic clinical information and tumor characteristics

A total of 113 NSCLC patients (82 male and 31 female) were enrolled in the study; the mean age of study participants was 57.2 ± 10.0 years (range, 35-78 years). There were 58 cases of lung adenocarcinoma, 52 cases of squamous cell carcinoma, and three cases of large cell carcinoma. Twenty-seven cases were well differentiated, 34 cases were moderately differentiated, and 52 cases were poorly differentiated. The cases were classified as stage I (n = 30), stage II (n = 48), stage III (n = 18), and stage IV (n = 17). Of the 113 cases, 67 had lymph node metastasis, according to surgery and pathology reports. Analyses of patient data after a 5-year follow-up showed that 77 patients had died; median survival was 21.0 months. As expected, median survival was longer for stage I-II patients (22.0 mo) than stage III-IV patients (13.0 mo; *P *= 0.001). There were no significant differences in survival according to gender, smoking history, histology, or grading. The clinical characteristics of study samples are shown in Table 1.

**Table 1:** Association of Oct-4 Expression with clinical features in NSCLC

Features	Total	Oct-4 expression *n *(%)^a^		*P*	*χ*^2^
				
		Negative	Positive		
Gender				0.330	0.674
Male	82	42 (51.2)	40 (48.8)		
Female	31	14 (45.2)	17 (54.8)		
Age (yr)^b^				0.348	1.082
≤58	54	24 (44.4)	30 (55.6)		
> 58	59	32 (54.2)	27 (45.8)		
Smoking				0.849	0.072
Yes	45	23 (51.1)	22 (48.9)		
No	68	33 (48.5)	35 (51.5)		
Histological type				< 0.001	13.637
Adenocarcinoma	58	21 (36.2)	37 (63.8)		
Squamous cell carcinoma	52	35 (67.3)	17 (32.7)		
Large cell carcinoma	3	0 (0.0)	3 (100.0)		
Histological differentiation				0.001	32.463
Well differentiated^c^	27	24 (88.9)	3 (11.1)		
Moderately differentiated	34	20 (58.8)	14 (41.2)		
Poorly differentiated^d^	52	12 (23.1)	40 (76.9)		
Adenocarcinoma				0.001	17.324
Well differentiated^c^	15	12 (80.0)	3 (20.0)		
Moderately differentiated	14	4 (28.6)	10 (71.4)		
Poorly differentiated	29	5 (17.2)	24 (82.8)		
Squamous cell carcinoma				0.001	16.780
Well differentiated	12	12 (100.0)	0 (0.0)		
Moderately differentiated	20	16 (80.0)	4 (20.0)		
Poorly differentiated	20	7 (35.0)	13 (65.0)		
Local advance				0.205	3.172
T_1_	30	17 (56.7)	13 (43.3)		
T_2_	48	26 (54.2)	22 (45.8)		
T_3_/T_4_	35	13 (37.1)	22 (62.9)		
Lymph node metastasis				0.466	1.529
N_0_	46	22 (47.8)	24 (52.2)		
N_1_	23	14 (60.9)	9 (39.1)		
N_2_	44	20 (45.5)	24 (54.5)		
Clinical stage				0.680	0.227
I/II	81	39 (48.1)	42 (51.9)		
III/IV	32	17 (53.1)	15 (46.9)		
MVD expression^a^				0.348	1.082
Positive	59	32 (54.2)	27 (45.8)		
Negative	54	24 (44.4)	30 (55.6)		
VEGF expression^a^				0.574	0.435
Positive	57	30 (52.6)	27 (47.4)		
Negative	56	26 (46.4)	30 (53.6)		
Ki-67 expression^a^				0.001	16.430
Positive	54	16 (29.6)	38 (70.4)		
Negative	59	40 (67.8)	19 (32.2)		

^a^Patients were divided according to the median values of immunohistochemical histoscores

^b^Patients were divided according to median age

^c^Bronchioloalveolar carcinoma was included in well differentiated

^d^Large cell carcinoma was included in poorly differentiated

### Association of Oct-4 expression with clinicopathological characteristics of NSCLC patients

**Figure 1:**
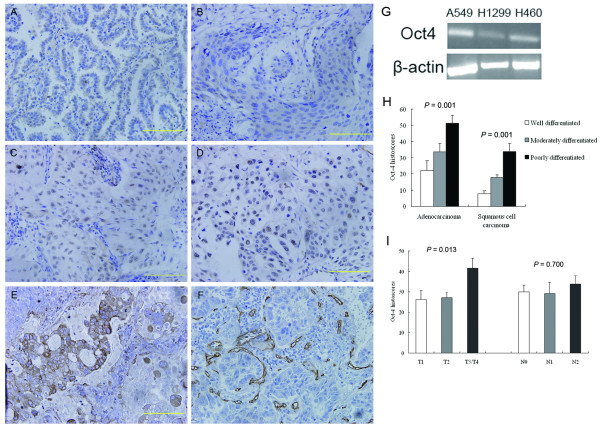
**Oct-4 expression in tissues of well-differentiated adenocarcinoma **(**A**), well-differentiated squamous cell carcinoma (**B**), poorly differentiated adenocarcinoma (**C**), and poorly differentiated squamous cell carcinoma (**D**), as well as VEGF staining (**E**) and MVD staining (**F**) were demonstrated immunohistologically. Quantification of Oct-4 expression (Oct-4 histoscore) with respect to differentiation status or tumor histology (**G**) and local advance or lymph nodes metastasis (**H**) is shown; 95% CIs are indicated.

Immunohistochemical analyses demonstrated that Oct-4 was expressed in 90.3% of samples (102/113 cases), with clear staining observed mostly in the nuclei of tumor cells; alveolar and bronchial epithelial cells in tumor-adjacent tissues were negative for Oct-4 staining (Figure 1). The histoscores of Oct-4 expression were variable among individual tumor samples. The mean Oct-4 histoscore was 31.32 ± 5.99 and the median histoscore was 25.80; this latter value was selected to categorize patients into Oct-4-positive (above the median) and -negative (below the median) groups. Among the 56 Oct-4-negative cases, 11 samples exhibited no Oct-4 staining. The associations of Oct-4-positive and -negative status with various clinical and pathological characteristics of NSCLC are shown in Table 1. Regarding to histoscores of Oct-4 staining, there was prominent discrepancy between adenocarcinoma and squamous cell carcinoma (39.40 ± 3.59 and 21.64 ± 2.47, *p *= 0.008). There was significant association of Oct-4 histoscores among well, moderated, and poor differentiation of tumor (15.69 ± 3.70, 24.27 ± 2.73, and 43.80 ± 3.49, *p *= 0.039), and quantification of staining also revealed that these associations differed markedly in adenocarcinoma or squamous cell carcinoma population (Figure 1H). There were no associations between Oct-4 expression and malignant local advance, lymph node metastasis, or TNM stage of disease (Figure 1I).

### Oct-4 expression in NSCLC cell lines

To better understand the expression status of Oct-4 in NSCLC, we examined the expression of Oct-4 in the NSCLC cell lines, A549, H460, and H1299. Oct-4 mRNA was detected in each of these cell lines (Figure 1G).

### Association of Oct-4 expression with malignant proliferation according to differences in VEGF-mediated angiogenesis

**Figure 2:**
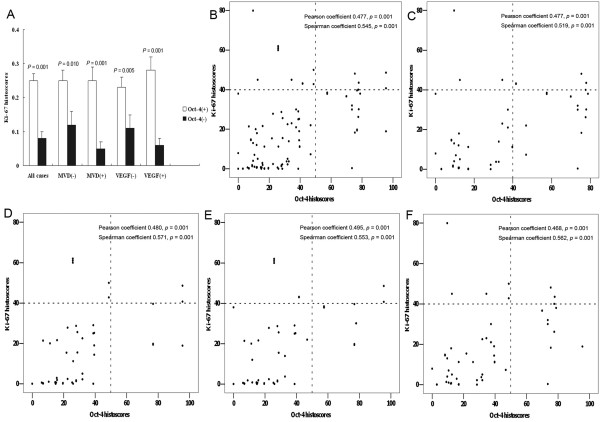
**Ki-67 expression histoscores were significantly different (ANOVA) according to different Oct-4 status in all cases, and in subsets of MVD-negative, MVD-positive, VEGF-negative, and VEGF-positive cases (**A**)**. All cases were divided into positive (above the median histoscore) and negative (below the median histoscore) groups. The association of Oct-4 staining with Ki-67 expression was positive in all cases (**B**), and in subsets of MVD-negative (**C**), MVD-positive (**D**), VEGF-negative (**E**), and VEGF-positive (**F**) cases. Statistical differences were calculated using Pearson and Spearman correlation analysis.

Intratumoral Ki-67 expression, a marker of malignant proliferation, varied according to Oct-4 phenotype in the population under study, with high Ki-67 expression showing a significant association with positive Oct-4 staining (Table 1). Quantification of staining revealed that this association differed markedly depending on Oct-4 histoscores (Figure 2A, p = 0.001) and showed that these two markers were positively correlated (Figure 2B). In MVD-negative and VEGF-negative subsets, intratumoral Ki-67 expression varied significantly according to Oct-4 phenotype (Figure 2A); Ki-67 (Figure 2C) and Oct-4 (Figure 2E) expression were also positively correlated in these subsets. These results suggest a prominent association of Oct-4 expression with malignant proliferation in NSCLC, especially in cases with weak VEGF-mediated angiogenesis.

### Association of Oct-4 expression with survival in all cases and in subsets of cases: univariate and multivariate analyses

**Figure 3:**
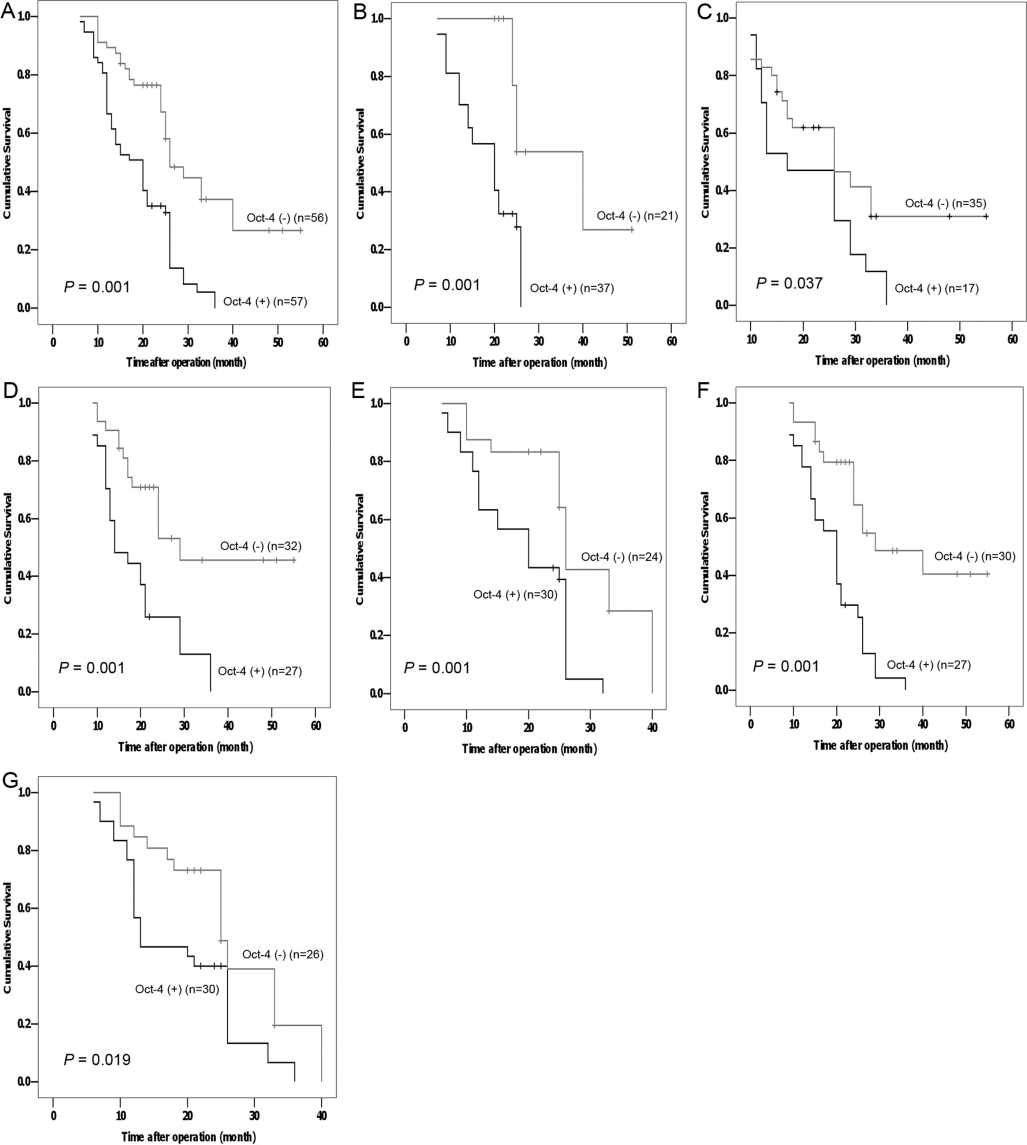
**Cumulative Kaplan-Meier survival curves based on the median values of Oct-4 immunochemical histoscores in NSCLC tissues are showed for all cases **(**A**), and for adenocarcinoma (**B**), squamous cell carcinoma (**C**), MVD-negative (**D**), MVD-positive (**E**), VEGF-negative (**F**), and VEGF-positive (**G**) cases. All cases were divided into positive (above the median histoscore) and negative (below the median histoscore) groups. Oct-4-positivity was associated with decreased overall survival in all subset. Statistical differences were calculated using log-rank comparisons.

The strength of associations between each individual predictor and overall survival was shown by univariate and multivariate analyses (Table 2). Oct-4 expression in tumor tissue and differentiation of tumor cells were strongly associated with cancer-associated death. Notably, an Oct-4 expression level less than the median histoscore (25.80) was associated with improved survival (HR, 1.011), whereas elevated Oct-4 expression was associated with shorter cumulative survival (*p *= 0.009). A Kaplan-Meier plot showed a prominent difference in survival estimates for patients with high versus low Oct-4 expression in tumor tissue; this difference corresponded to a median survival of 18.2 ± 6.0 months for patients with high Oct-4 expression compared with a median survival of more than 24.7 ± 9.1 months for patients with low Oct-4 expression (Figure 3A). More importantly, significant differences were also found in the adenocarcinoma subset (17.7 ± 9.1 vs. 27.3 ± 9.6 months; Figure 3B) and the squamous cell carcinoma subset (20.7 ± 9.5 vs. 23.2 ± 10.8 months; Figure 3C). When all predictors were included in a Cox model, Oct-4 expression retained its prognostic significance for overall survival. Hence, a low level of Oct-4 expression in tumor tissue predicted improved survival in NSCLC patients.

**Table 2:** Univariate and multivariate analyses of individual variables for correlations with overall survival: cox proportional hazards model

Variables	Univariate			Multivariate		
	
	HR	95%CI	*P*	HR	95%CI	*P*
Age	0.988	0.969-1.008	0.231	1.001	0.978-1.025	0.922
Gender	0.852	0.517-1.405	0.530	0.525	0.305-0.906	0.121
Smoking	1.179	0.740-1.880	0.489	1.277	0.743-2.195	0.376
Histological type	1.087	0.697-1.695	0.713	1.168	0.706-1.932	0.546
Histological differentiation	3.727	2.139-6.495	< 0.001	3.666	1.937-6.939	0.001
Local advance	1.282	0.920-1.731	0.149	1.222	0.928-1.609	0.153
Lymph node metastasis	1.487	1.148-1.927	0.003	1.042	0.743-1.461	0.813
Oct-4 expression	1.105	1.007-1.024	< 0.001	1.011	1.003-1.020	0.009

Age	0.990	0.963-1.018	0.482	1.014	0.978-1.051	0.450
Gender	0.786	0.408-1.512	0.470	0.296	0.087-1.008	0.052
Smoking	1.231	0.646-2.346	0.527	0.733	0.237-2.265	0.590
Histological type	0.785	0.408-1.512	0.470	0.869	0.386-1.956	0.735
Histological differentiation	1.428	0.701-2.910	0.327	1.418	0.591-3.405	0.434
Local advance	1.191	0.780-1.817	0.418	0.934	0.560-1.558	0.793
Lymph node metastasis	1.217	0.833-1.778	0.310	1.560	0.976-2.495	0.063
Oct-4 expression	1.014	1.002-1.025	0.021	1.024	1.007-1.042	0.005

Age	0.994	0.965-1.024	0.688	1.005	0.967-1.044	0.801
Gender	0.790	0.395-1.580	0.505	0.401	0.166-0.966	0.096
Smoking	1.232	0.635-2.389	0.537	0.921	0.382-2.219	0.855
Histological type	1.439	0.767-2.700	0.257	1.247	0.598-2.600	0.556
Histological differentiation	1.925	0.934-3.969	0.076	1.962	0.791-4.868	0.146
Local advance	1.313	0.822-2.099	0.254	1.231	0.743-2.042	0.420
Lymph node metastasis	1.415	0.953-2.103	0.086	1.472	0.933-2.323	0.097
Oct-4 expression	1.010	0.999-1.022	0.018	1.011	0.998-1.024	0.042

Variable 1, Oct-4 expression was an independent prognostic factor, adjusted by histological differentiation, in all cases

Variable 2, Oct-4 expression was an independent factor in MVD-negative cases

Variable 3, Oct-4 expression was an independent factor in VEGF-negative cases

Abbreviations: *HR*, hazard ratio; *CI*, confidence interval

In order to observe the contribution of Oct-4 to overall survival in patients in which VEGF-mediated angiogenesis was disabled, we also performed univariate and multivariate analyses in MVD-negative and VEGF-negative subsets (Table 2). Notably, an Oct-4 expression level less than the median histoscore was associated with improved survival, whereas elevated Oct-4 expression was associated with shorter cumulative survival in both the MVD-negative subset (HR, 1.024, *p *= 0.005) and the VEGF-negative subset (HR, 1.011, *p *= 0.042). Further, a Kaplan-Meier plot showed a prominent difference in survival estimates for patients in the MVD-negative subset, where the median survival for patients with high Oct-4 expression was 18.5 ± 7.6 months compared with a median survival of more than 24.3 ± 8.3 months for patients with low Oct-4 expression (Figure 3D). Similar differences were found for patients in the VEGF-negative subset; here the median survival for patients with high Oct-4 expression was 17.5 ± 6.1 months compared with a median survival of more than 21.9 ± 7.5 months for patients with low Oct-4 expression (Figure 3F). Hence, Oct-4 expression retained its prognostic significance for overall survival in NSCLC patients with weak VEGF-mediated angiogenesis.

## Discussion

Although Oct-4 has been detected in various carcinomas, including breast cancer [9], bladder cancer [10], prostate cancer [11] and lung adenocarcinoma [20], the precise role of this stem cell marker in maintaining the survival of cancer cells is unclear. Sustained expression of Oct-4 in epithelial tissues has been shown to lead to dysplastic changes through inhibition of cellular differentiation, similar to its action in some progenitor cells, suggesting that Oct-4 may play an important role in the genesis of tumors [21]. However, the mechanisms by which Oct-4 acts during tumor progression have remained poorly understood. Accordingly, we examined the behavior of Oct-4 in primary NSCLC tissues, focusing on the associations of Oct-4 expression with clinicopathological features and markers of tumor-induced angiogenesis. Important in this context is the observation that, after disregarding nonangiogenic subsets of NSCLC (which tend to obscure the association of Oct-4 with tumor angiogenesis), a subset of NSCLC tumors does not induce angiogenesis, but instead co-opts the normal vasculature for further growth.

On the basis of the previous finding that Oct-4 may be a major contributor to the maintenance of self-renewal in embryonic stem cells, we investigated the association of Oct-4 expression with self-renewal of NSCLC cells. The immunohistochemical analyses presented here showed clear Oct-4 staining in most sections, and RT-PCR showed Oct-4 mRNA in all NSCLC cell lines. Our data extend the previous report of Oct-4 overexpression in lung adenocarcinoma [20], providing the first demonstration that Oct-4 is also present in lung squamous cell carcinoma specimens, exhibiting an apparent difference in the degree of expression among sections analyzed. One possible explanation for these findings is that the genesis of lung adenocarcinoma and squamous cell carcinoma may be different. The former arises from mucous glands or the cells of bronchoalveolar duct junction and the latter grows most commonly in or around major bronchi. Further studies designed to address the relationship between Oct-4 expression in endothelial precursors and the sites of origin of adenocarcinoma and squamous cell carcinoma are required to confirm this. Our data also showed that the degree of immunohistochemical staining was positively correlated with poor differentiation of tumor cells and Ki-67 expression; this latter marker provides an opportunity to analyze the proliferative cell fraction in preserved tumor specimens. High levels of Oct-4 have been shown to increase the malignant potential of tumors, whereas inactivation of Oct-4 induces a regression of the malignant component [22]; moreover, knockdown of Oct-4 expression in lung cancer cells has been shown to facilitate differentiation of CD133-positive cells into CD133-negative cells [23]. These findings, taken together with our data, indicate that overexpression of Oct-4 in NSCLC tissues may maintain the poorly differentiated state by contributing to tumor cell proliferation. On the other hand, down-regulation of Oct-4 expression has been shown to induce apoptosis of tumor-initiating-cell-like cells through an Oct-4/Tcl1/Akt1 pathway, implying that Oct-4 might maintain the survival of tumor-initiating cells, at least in part, by inhibiting apoptosis [13]. Whether an Oct-4-dependent pathway modulates apoptosis in clinical NSCLC samples or NSCLC cell lines has not yet been tested.

Previous reports have indicated that tumor-induced angiogenesis is important in maintaining the poorly differentiated state and promoting metastasis in NSCLC [23,24]. In our study, we observed an association of Oct-4 expression in NSCLC specimens with some features of tumor-induced angiogenesis, but the investigation revealed no prominent linkage between Oct-4 expression and neovascularization (defined by CD34 and VEGF-A expression). However, Passalidou and Pezzella have previously described a subset of NSCLC without morphological evidence of neo-angiogenesis. In these tumors, alveoli are filled with neoplastic cells and the only vessels present appeared to belong to the trapped alveolar septa; moreover, tumors with normal vessels and no neo-angiogenesis seemed resistant to some anti-angiogenic therapies [16,17]. In this context, we observed an association of Oct-4 expression with tumor cell proliferation in patients with weak VEGF-mediated angiogenesis, including MVD-negative and VEGF-negative subsets, indicating that Oct-4 still plays an important role in cell proliferation in NSCLC tumors, even those with weak MVD or VEGF status. Whether Oct-4 expression contributes to resistance to anti-angiogenic therapy thus warrants additional research attention.

Although recent reports have also shown that Oct-4 is re-expressed in different human carcinomas, implicating Oct-4 as a potential diagnostic marker in malignancy [25,26], whether Oct-4 expression can be used as a diagnostic tool to monitor the clinical prognosis of NSCLC patients has not been previously substantiated. An analysis of our follow-up data designed to definitively assess the effect of Oct-4 immunohistochemical expression on the prognosis of NSCLC patients showed that the post-operative survival duration of patients with high Oct-4 expression was notably shorter than that of patients with low expression. These results indicate that overexpression of Oct-4 has a detrimental effect on prognosis, and further demonstrates that Oct-4 expression may be correlated with the malignant behavior of tumors during NSCLC progression. A combined genomic analysis of the Oct-4/SOX2/NANOG pathway has recently demonstrated high prognostic accuracy in studies of patients with multiple tumor types [27]. Similarly, multivariate analyses of the data presented here demonstrated that Oct-4 expression is an independent factor whose expression might indicate poor prognosis of patients with NSCLC, generally, as well in NSCLC patient subsets, especially those with weak or no neovascularization. A detailed investigation of the association of Oct-4 expression with treatment response, particularly a characterization of the molecular phenotype of tumors following downregulation of Oct-4, would provide further support for this interpretation.

## Conclusion

In summary, a multivariate analysis demonstrated that Oct-4 expression was an independent predictor of overall survival, suggesting that Oct-4 may be useful as a molecular marker to assess the prognosis of patients with primary NSCLC, especially those without prominent neovascularization. In patients without prominent tumor-induced angiogenesis, Oct-4-overexpressing cells in primary NSCLC tissue represent a reservoir of tumor cells with differentiation potential; moreover, Oct-4 may maintain tumor cells in a poorly differentiated state through a mechanism that depends on promoting cell proliferation. The molecular mechanisms by which Oct-4 sustains the self-renewal capacity of tumor cells, especially those with poor neovascularization status, are poorly understood and are the focus of our future studies. Developing strategies to inhibit Oct-4 during tumor progression may have positive prognostic implications in primary NSCLC patients.

## Abbreviations

NSCLC: Non-small cell lung cancer; Oct-4: Octamer 4; VEGF: Vascular endothelial growth factor; CI: Confidence interval; HR: Hazard ratio; MVD: Microvessel density; TNM: Tumor-node-metastases.

## Competing interests

The authors declare that they have no competing interests.

## Authors' contributions

ZC and TW conceived the study, participated in the analysis of NSCLC specimens and cell lines, and drafted the manuscript. TW, LC, CS, BZ, and YL managed the histopathological analysis of tumor samples and performed the RT-PCR analysis of cell lines. HL participated in patient enrollment and participated in the preparation of the manuscript. ZC, TW, and AP coordinated the study and drafted the manuscript. All authors have read and approved the final manuscript.

## Acknowledgements

Grant support: This work was supported by grants from the National Basic Research Program of China (973 Program, No. 2008CB517406), the National Natural Science Foundation of China (No. 30671023, 30971675, 30900729), and the Key Scientific and Technological Projects of Guangdong Province (No. 2007A032100003).

## References

[B1] Ozols RF Herbst RS Colson YL Gralow J Bonner J Curran WJ Jr Eisenberg BL Ganz PA Kramer BS Kris MG Markman M Mayer RJ Raghavan D Reaman GH Sawaya R Schilsky RL Schuchter LM Sweetenham JW Vahdat LT Winn RJ American Society of Clinical Oncology: Clinical cancer advances 2006: major research advances in cancer treatment, prevention, and screening-a report from the American Society of Clinical Oncology J Clin Oncol 2007 25 146 162 17158528 10.1200/JCO.2006.09.7030

[B2] D'Addario G Felip E Non-small-cell lung cancer: ESMO clinical recommendations for diagnosis, treatment and follow-up Ann Oncol 2009 20 Suppl 4 68 70 19454467 10.1093/annonc/mdp132

[B3] Burdon T Smith A Savatier P Signalling, cell cycle and pluripotency in embryonic stem cells Trends Cell Biol 2002 12 432 438 10.1016/S0962-8924(02)02352-8 12220864

[B4] Niwa H Miyazaki J Smith AG Quantitative expression of Oct-3/4 defines differentiation, dedifferentiation or self-renewal of ES cells Nat Genet 2000 24 372 376 10.1038/74199 10742100

[B5] Patrawala L Calhoun T Schneider-Broussard R Li H Bhatia B Tang S Reilly JG Chandra D Zhou J Claypool K Coghlan L Tang DG Highly purified CD44+ prostate cancer cells from xenograft human tumors are enriched in tumorigenic and metastatic progenitor cells Oncogene 2006 25 1696 1708 10.1038/sj.onc.1209327 16449977

[B6] Matoba R Niwa H Masui S Ohtsuka S Carter MG Sharov AA Ko MS Dissecting Oct3/4-regulated gene networks in embryonic stem cells by expression profiling PLoS One 2006 1 e26 10.1371/journal.pone.0000026 17183653 PMC1762406

[B7] Park IH Zhao R West JA Yabuuchi A Huo H Ince TA Lerou PH Lensch MW Daley GQ Reprogramming of human somatic cells to pluripotency with defined factors Nature 2008 451 141 146 10.1038/nature06534 18157115

[B8] Brehm A Ohbo K Zwerschke W Botquin V Jansen-Dürr P Schöler HR Synergism with germ line transcription factor Oct-4: viral oncoproteins share the ability to mimic a stem cell-specific activity Mol Cell Biol 1999 19 2635 2643 10082529 10.1128/mcb.19.4.2635 PMC84056

[B9] Gu G Yuan J Wills M Kasper S Prostate cancer cells with stem cell characteristics reconstitute the original human tumor in vivo Cancer Res 2007 67 4807 4815 10.1158/0008-5472.CAN-06-4608 17510410

[B10] Ezeh UI Turek PJ Reijo RA Clark AT Human embryonic stem cell genes OCT4, NANOG, STELLAR, and GDF3are expressed in both seminoma and breast carcinoma Cancer 2005 104 2255 2265 10.1002/cncr.21432 16228988

[B11] Atlasi Y Mowla SJ Ziaee SA Bahrami AR OCT-4, an embryonic stem cell marker, is highly expressed in bladder cancer Int J Cancer 2007 120 1598 1602 10.1002/ijc.22508 17205510

[B12] Shin S Mitalipova M Noggle S Tibbitts D Venable A Rao R Stice SL Long-term proliferation of human embryonic stem cell-derived neuroepithelial cells using defined adherent culture conditions Stem Cells 2006 24 125 138 10.1634/stemcells.2004-0150 16100006

[B13] Hu TS Liu SR Breiter DR Wang F Tang Y Sun S Octamer 4 small interfering RNA results in cancer stem cell-like cell apoptosis Cancer Res 2008 68 6533 6540 10.1158/0008-5472.CAN-07-6642 18701476

[B14] Hu J Qin K Zhang Y Gong J Li N Lv D Xiang R Tan X Downregulation of transcription factor Oct4 induces an epithelial-to-mesenchymal transition via enhancement of Ca2+ influx in breast cancer cells Biochem Biophys Res Commun 2011 411 4 786 791 10.1016/j.bbrc.2011.07.025 21798248

[B15] Karoubi G Gugger M Schmid R Dutly A Oct4 expression in human non-small cell lung cancer: implications for therapeutic intervention Interact Cardiovasc Thorac Surg 2009 8 393 397 10.1510/icvts.2008.193995 19126554

[B16] Passalidou E Trivella M Singh N Ferguson M Hu J Cesario A Granone P Nicholson AG Goldstraw P Ratcliffe C Tetlow M Leigh I Harris AL Gatter KC Pezzella F Vascular phenotype in angiogenic and non-angiogenic lung non-small cell carcinomas Br J Cancer 2002 86 244 249 10.1038/sj.bjc.6600015 11870514 PMC2375177

[B17] Pezzella F Pastorino U Tagliabue E Andreola S Sozzi G Gasparini G Menard S Gatter KC Harris AL Fox S Buyse M Pilotti S Pierotti M Rilke F Non-small-cell lung carcinoma tumor growth without morphological evidence of neo-angiogenesis Am J Pathol 1997 151 1417 1423 9358768 PMC1858069

[B18] Sobin LH Wittekind CH International Union Against Cancer (UICC) TNM Classification Of Malignant Tumors 2002 6 New York: Wiley-Liss 99 103

[B19] Travis WD Brambilla E Muller-Hermelink HK WHO classification of tumors. Pathology and Genetics. Tumors of lung, pleura, thymus and heart 2004 Lyon: IARC Press 9 124

[B20] Teng Y Wang X Wang Y Ma D Wnt/beta-catenin signaling regulates cancer stem cells in lung cancer A549 cells Biochem Biophys Res Commun 2010 392 373 379 10.1016/j.bbrc.2010.01.028 20074550

[B21] Hochedlinger K Yamada Y Beard C Jaenisch R Ectopic expression of Oct-4 blocks progenitor-cell differentiation and causes dysplasia in epithelial tissues Cell 2005 121 465 477 10.1016/j.cell.2005.02.018 15882627

[B22] Gidekel S Pizov G Bergman Y Pikarsky E Oct-3/4 is a dose-dependent oncogenic fate determinant Cancer Cell 2003 4 361 370 10.1016/S1535-6108(03)00270-8 14667503

[B23] Hirakawa S Kodama S Kunstfeld R Kajiya K Brown LF Detmar M VEGF-A induces tumor and sentinel lymph node lymphangiogenesis and promotes lymphatic metastasis J Exp Med 2005 201 1089 1099 10.1084/jem.20041896 15809353 PMC2213132

[B24] Kadota K Huang CL Liu D Ueno M Kushida Y Haba R Yokomise H The clinical significance of lymphangiogenesis and angiogenesis in non-small cell lung cancer patients Eur J Cancer 2008 44 1057 1067 10.1016/j.ejca.2008.03.012 18396396

[B25] Chen YC Hsu HS Chen YW Tsai TH How CK Wang CY Hung SC Chang YL Tsai ML Lee YY Ku HH Chiou SH Oct-4 expression maintained cancer stem-like properties in lung cancer-derived CD133-positive cells PLoS One 2008 3 e2637 10.1371/journal.pone.0002637 18612434 PMC2440807

[B26] Sung MT Jones TD Beck SD Foster RS Cheng L OCT4 is superior to CD30 in the diagnosis of metastatic embryonal carcinomas after chemotherapy Hum Pathol 2006 37 662 667 10.1016/j.humpath.2006.01.019 16733205

[B27] Glinsky GV "Stemness" genomics law governs clinical behavior of human cancer: implications for decision making in disease management J Clin Oncol 2008 26 2846 2853 10.1200/JCO.2008.17.0266 18539963

